# Prevalence and type of artefact with spectral domain optical coherence tomography macular ganglion cell imaging in glaucoma surveillance

**DOI:** 10.1371/journal.pone.0206684

**Published:** 2018-12-05

**Authors:** Mona S. Awadalla, Jude Fitzgerald, Nicholas H. Andrew, Tiger Zhou, Henry Marshall, Ayub Qassim, Mark Hassall, Robert J. Casson, Stuart L. Graham, Paul R. Healey, Ashish Agar, Anna Galanopoulos, Simon Phipps, Angela Chappell, John Landers, Jamie E. Craig

**Affiliations:** 1 Flinders University, Department of Ophthalmology, South Australia, Australia; 2 University of Adelaide, Discipline of Ophthalmology & Visual Sciences, Adelaide, Australia; 3 Faculty of Medicine and Health Sciences, Macquarie University, Sydney, Australia; 4 University of Sydney Discipline of Ophthalmology, Sydney, Australia; 5 Centre for Vision Research, Westmead Institute for Medical Research, University of Sydney, Sydney, Australia; 6 Marsden Eye Specialists, Sydney, Australia; 7 Department of Ophthalmology, Prince of Wales Hospital, University of New South Wales, Sydney, Australia; 8 Goodwood Eye Centre, Millswood, South Australia; University of Houston, UNITED STATES

## Abstract

**Purpose:**

The ganglion cell analysis (GCA) of the CIRRUS^TM^ HD-OCT (Carl Zeiss, Meditec; Dublin, CA) provides measurement of the macular ganglion cell-inner plexiform layer (GCIPL) thickness. This study determined the frequency of scan artefacts and errors in GCIPL imaging in individuals undergoing HD-OCT surveillance for glaucoma.

**Method:**

A total of 1439 eyes from 721 subjects enrolled in a prospective study assessing predictors of glaucoma progression underwent macular GCIPL imaging with the CIRRUS HD-OCT at recruitment. The prevalence of acquisition errors, segmentation errors, and co-morbid macular pathology was determined.

**Results:**

A total of 87 (6.0%) of the 1439 scans had either acquisition errors, segmentation artefacts, or other macular pathology. The most common co-morbid macular pathology was epiretinal membrane in 2.2% of eyes.

**Conclusion:**

The macular GCIPL scan was artefact free in 94% of eyes. However, epiretinal membrane and high myopia can cause scan artefact and should be considered when interpreting the results.

## Introduction

The development of optical coherence tomography (OCT) and its refinement over the past two decades has had a profound impact on glaucoma diagnosis and management. Analysis of the circumpapillary retinal nerve fibre layer (cRNFL) thickness is an important tool in diagnosis and monitoring. [1] Improvement in OCT technology from time domain to spectral domain ocular coherence tomography (SD-OCT) has allowed higher resolution imaging and accurate segmentation of the retinal layers at the macula. In addition, ganglion cell analysis (GCA) software provides automated measurement of the thickness of the ganglion cell layer and inner plexiform layer (GCIPL) and has similar performance to cRNFL measurements in detecting glaucomatous structural change. [2, 3]

GCIPL analysis with the SD-OCT may be complicated by co-existing macular pathology and scan artefact. Macular and vitreo-macular pathologies can interfere with accurate segmentation of the retinal layers and measurement of GCIPL thickness. For this reason, studies assessing the performance of macula SD-OCT scans in glaucoma have usually excluded subjects with high refractive errors, age-related macular degeneration or pathology at the vitreo-macular interface. However, macular abnormalities, particularly epiretinal membranes are common in the ageing population [4], and individuals with both glaucoma and macular pathology are frequently encountered in clinical practice. Recent studies using GCIPL analysis with the CIRRUS^TM^ OCT have excluded 1.8% to 5.15%. of scans due to machine segmentation or acquisition error. [2, 3, 5, 6]

The aim of this study was to identify the prevalence and the type of errors that would be identified in GCIPL scans in a large “real-world” population of individuals undergoing SD-OCT surveillance for glaucoma.

## Methods

The “Progression Risk Of Glaucoma; RElevant SNPs with Significant Association (PROGRESSA) Study”, is an ongoing prospective 5-year study investigating predictors of progression in a cohort of patients with suspect glaucoma or early manifest glaucoma. Exclusion criteria included visual acuity of less than 6/18 in the affected eye, and visual field defect not attributable to glaucoma. The study followed the tenets of the Declaration of Helsinki. Ethical approval for the study was granted by the Human Ethics Committee of the Flinders Medical Centre, South Australia. The current study is an interim report utilizing data currently available from the PROGRESSA study. A participant information sheet and verbal detailed explanation were provided to all participants, and written informed consents were obtained.

At recruitment, all subjects enrolled in the PROGRESSA study had pupil dilation with tropicamide 1% before the Cirrus HD-OCT (Carl Zeiss Meditec, Dublin, CA) macular cube 512x128 scans were performed.

These scans were analyzed with the commercially available GCA software, which measures the GCIPL thickness within an elliptical annulus centered on the fovea. Scans not centered on the fovea were manually re-centered prior to analysis.

Baseline HD-OCT imaging was performed on a total of 721 individuals, comprising 253 patients with early manifest glaucoma and 468 glaucoma suspects. This provided a total of 1439 eyes with baseline HD-OCT macular GCA images for assessment. Glaucoma suspects were defined as patients who do not demonstrate a glaucomatous visual field defect, using the Hodapp-Parrish-Anderson (HPA) criteria, [7] on a reliable Humphrey Visual Field 24–2 SITA Standard test, but who have an optic nerve head or neuro-retinal rim appearance suspicious of glaucoma. Suspicious optic nerve head or neuroretinal rim was defined by The Disc Damage Likelihood Scale (DDLS) [8] of stage 1 or greater in the absence of a glaucomatous field defect as defined above. Early manifest glaucoma was defined as patients who had reproducible visual field loss in glaucomatous regions with mean deviation better than -6dB. A baseline field was considered abnormal if the glaucoma hemifield test (GHT) was outside normal limits, corrected pattern standard deviation (PSD) of P<0.05 or there was a cluster of ≥3 contiguous points on the pattern deviation plot depressed below 5% level, at least one of which is below 1% level, as per HPA criteria. A confirmatory visual field within 6-months demonstrating a cluster in the same region was required. Alternatively, if the confirmatory visual field had an abnormal GHT or PSD (as defined above), the cluster of contiguous points only needed to be below 5% level.

Scans were independently reviewed by two of our glaucoma consultants (JEC and JL) who assessed the horizontal B-scan image, a cross-sectional image of the morphology of the retina [9], intersecting the fovea for any evidence of acquisition error, segmentation artefact, or identifiable co-morbid macular pathology. Signal strength was also recorded and scans with signal strength less than 6/10 were categorized as “poor quality”. Acquisition errors were defined either as errors of scan alignment by the operator or eye movement, resulting in part of the macula lying anterior or posterior to the volume of space imaged by the macular cube. Machine segmentation errors were defined as incorrect identification of the outer border of the RNFL or outer border of the inner plexiform layer by the GCA software, despite good scan quality.

## Results

The mean age of the participants was 64.7 (SD 10.1) years-old and 57% were females. Of the 1439 eyes scanned, 87 scans (6.0%) had either a signal strength of less than 6, scan acquisition error, machine segmentation error, or identifiable co-morbid macular pathology Table 1. The most common abnormality was epiretinal membrane, affecting 2.2% of eyes. Each of the other abnormalities occurred in less than 1% of scans.

The mean age of participants who presented with GCIPL scan errors was 71.5 (SD 8.8) years old. Out of the 87 eyes; 39 had early manifest glaucoma, and 48 were glaucoma suspects. In our study, age and disease stage did not show any association with the prevalence of GCIPL errors S1 Table. 22 eyes showed errors on RNFL thickness scans on the same day of the GCIPL scan. Out of the 22 eyes, 6 had acquisition artefacts which were corrected, and the remaining 16 eyes had structural abnormalities which resulted in abnormal RNFL scans such as ERM or high myopia.

**Table 1:** The prevalence of identified scan errors and identifiable co-morbid macular pathology in the PROGRESSA population.

Error type	Number of eyes	% of scans
Signal strength <6/10	11	0.8
Acquisition error	2	0.1
Machine segmentation error	12	0.8
Obscuration eg floaters	11	0.8
Epiretinal membrane*	32	2.2
Vitreo-macular interface abnormality	5	0.2
Macular schisis or non-full thickness macular hole	4	0.3
Drusen / age related maculopthy	2	0.1
Myopic error (including staphyloma)*	11	0.7
TOTAL	87	6.0

*3 eyes are included in both the myopic error and epiretinal membrane categories.

### Poor scan signal strength

11 eyes were found to have signal strength less than 6/10 due to cataract and ocular surface disease (i.e dry eye). In many cases the signal strength can be improved by repeating the scan. Where scans from subsequent visits were available, 9 of 11 poor quality scans were improved to an acceptable level with a signal strength ≥ 6/10.

### Acquisition errors

**Fig 1:**
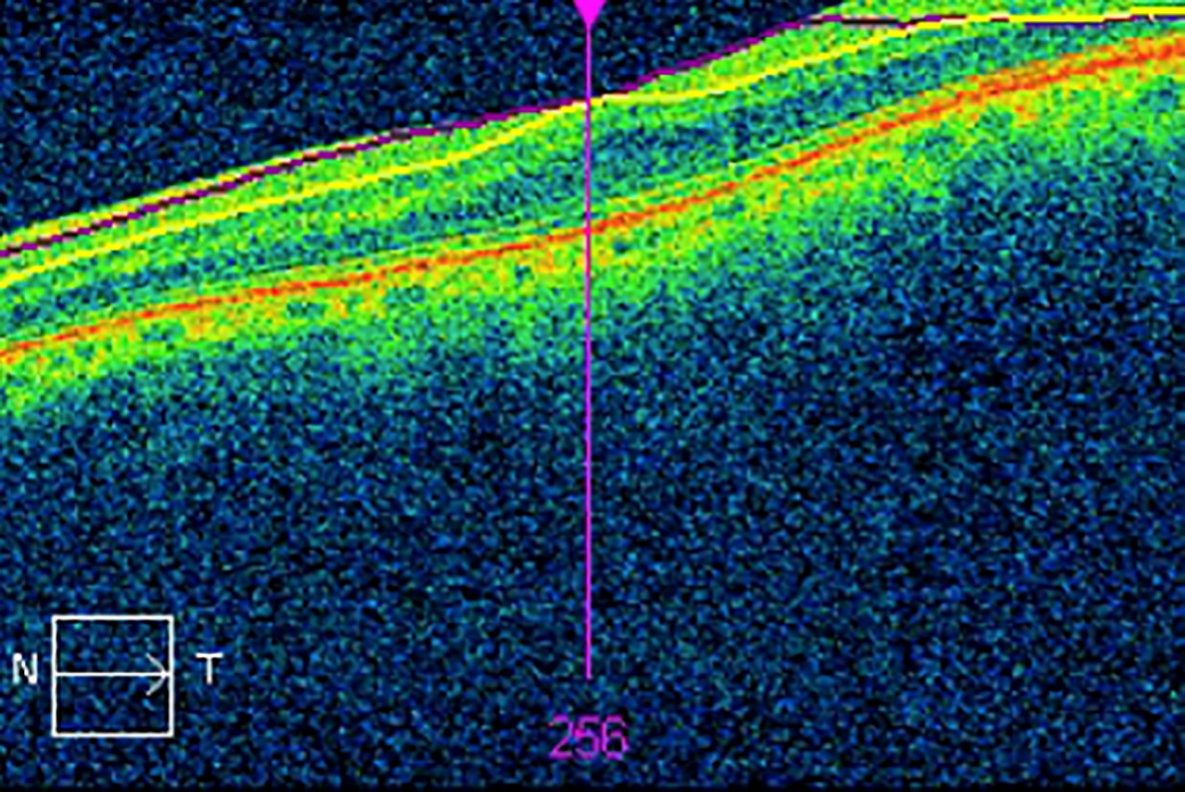
An OCT scan demonstrating an acquisition error due to poor image alignment by the operator. Part of the macula is anterior to the volume of space imaged by the macular cube. This results in missing data from the Ganglion Cell Analysis.

This is easily corrected if the error is identified and the scan is repeated Fig 1. However, eyes with high axial myopia and posterior staphylomas can be difficult to capture within the macular cube.

### Machine segmentation errors

**Fig 2:**
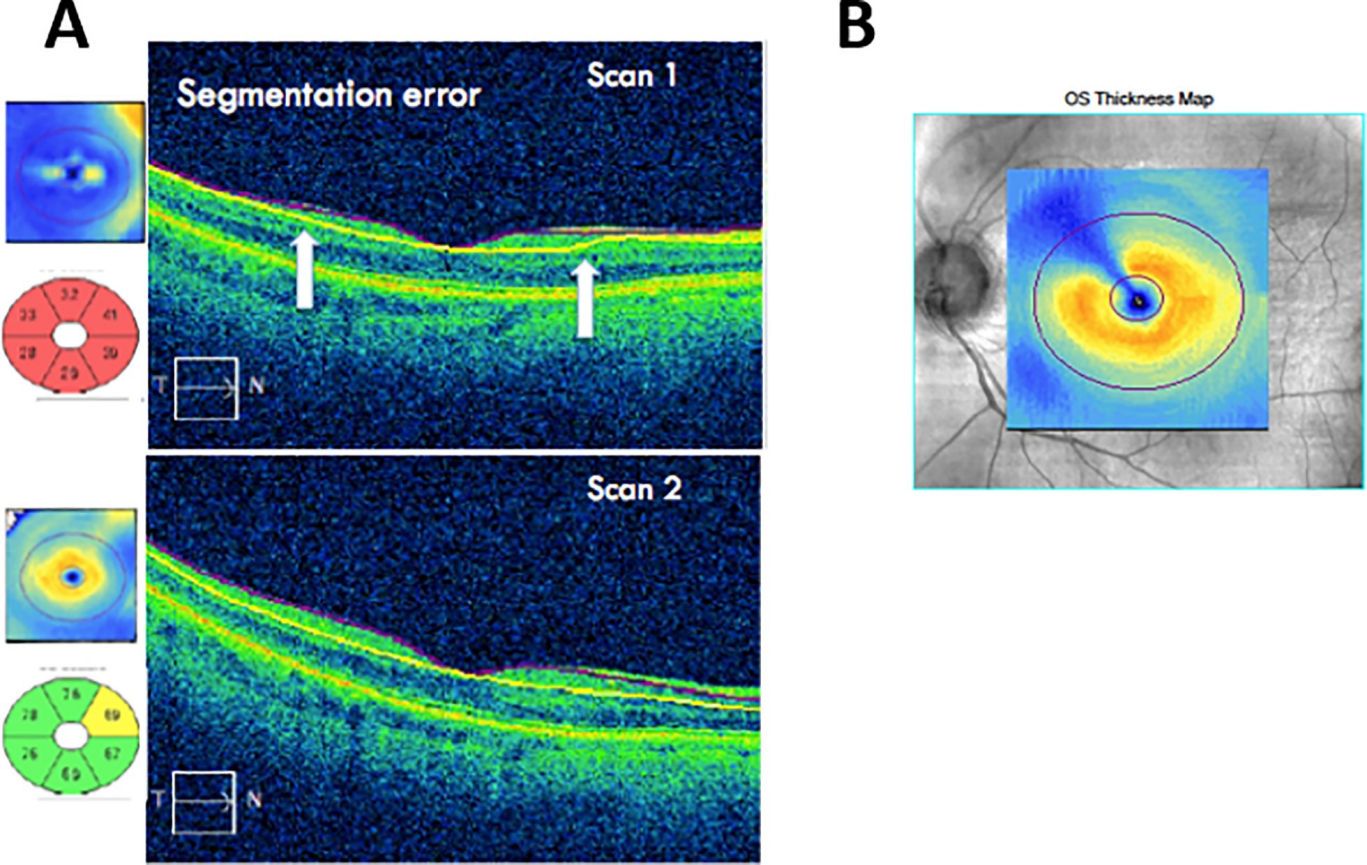
**Segmentation error** (A) machine segmentation error (Scan 1) with white arrows showing loss of accurate segmentation. Repeat scanning at a subsequent visit (Scan 2) shows correct segmentation. (B) focal radial segmentation defect.

Twelve eyes had machine segmentation error, in which 6 were readily evident as a focal, radial wedge of thinning on the macular GCIPL thickness map Fig 2, without obvious pathology on the OCT B-scan. All six scans were segmented correctly on subsequent scanning, confirming the macular GCIPL thinning was due to segmentation error rather than a pathological cause.

### Obscuration

The presence of vitreous floaters can focally obscure the signal from the retina, leading to an inability to segment the retinal layers. The effect on the scan will depend on the location of the floater at the time of the scan. If the floater is outside the elliptical annulus scan area, it has negligible effect on the segmentation.

### Co-morbid macular pathology

**Fig 3:**
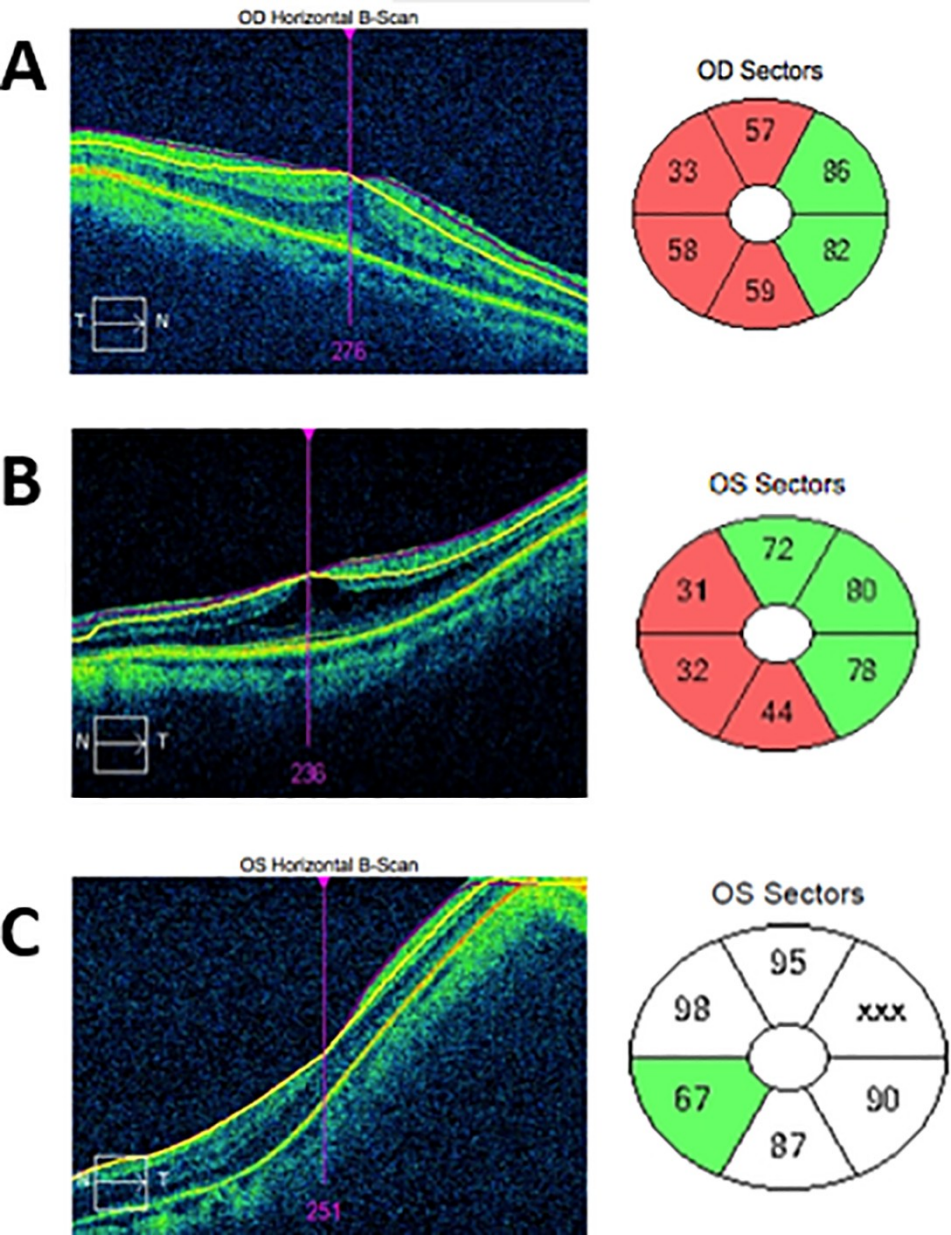
Pathological errors. (A) Epiretinal membrane showing horizontal B scan with attempted machine segmentation, and resulting abnormal Ganglion cell complex plot. (B) Macular schisis, with subsequent poor analysis of ganglion cell layering. Poor quality scan. (C) Myopic staphyloma.

The most common identifiable error on horizontal B-scans of the macula was epiretinal membrane, with 32 eyes (2.2%) identified Fig 3A. Other macular pathologies were identified in nine cases through horizontal OCT B-scan. These include three cases of vitreomacular traction, four of macular schisis Fig 3B, and two of drusen.

### Myopic staphyloma

130 eyes had myopic refraction of at least 3 diopters. Of these, 11 (0.7%) eyes had severe artefact secondary to posterior myopic staphyloma; of which 4 eyes had moderate myopia, with a refractive error of magnitude between 3 to 6 diopters, and seven eyes had high myopia (< 6 diopters of myopic correction) Fig 3C.

## Discussion

The utility of GCIPL analysis in glaucoma diagnosis and surveillance is becoming increasingly recognized. Mwanza *et al*. reported that GCIPL analysis has a high diagnostic performance for detecting early perimetric glaucoma and is consistent with changes in the RNFL parameters [10]. These findings were supported by Nouri-Mahdavi *et al*., who reported that combining both GCIPL and RNFL data was synergistic for diagnosing early glaucoma.[11]

However, the presence of HD-OCT scan artefacts is a recognized problem that requires vigilance when interpreting images. In a retrospective chart review evaluating the impact of artefacts on foveal thickness measurements in 98 eyes, Han *et al*. reported that 85% of Cirrus HD-OCT macula scans had at least 1 artefact.[12] Asrani *et al*. reported that 19.9% of the SD OCT RNFL scans contained artefacts mainly related to the presence of epiretinal membranes (ERM). [13] Additionally, a study on 1188 individuals with glaucoma reported that 46.3% of RNFL scans have at least 1 artefact. [14] In the present study, we found identifiable errors in 6.0% of GCA scans performed on a cohort of glaucoma suspects and individuals with early manifest glaucoma.

Although scan errors and co-morbid macular pathology can confound ganglion cell analysis, the problem can often be easily rectified. Some scan errors could be improved simply by re-scanning; however, the error needs to be recognized by the operator. The ability to identify and classify any type of error is important, as re-scanning the macula can correct poor image quality, acquisition and segmentation errors. These issues need to be considered during assessment of the OCT scans. In our study, poor signal scans were often secondary to coexisting conditions such as cataract or dry eye (common in patients on topical glaucomatous medication), [15] which agrees with previous published literature on the effect of lens opacity and ocular surface disease on the quality of OCT retinal nerve fibre layer scans. [10, 16, 17]

Although we had stringent criteria to exclude patients with a central field defect due to macular pathologies from our prospective PROGRESSA cohort, a small but important percentage of scans showed an abnormal GCIPL result due to factors other than glaucoma. It included patients with milder macular pathology not significantly affecting their vision. Therefore, clinicians should consider a macular OCT as part of glaucoma surveillance to rule out co-existing macular pathology that may cause artefacts. For an example, epiretinal membrane often causes the ganglion cell complex to appear thinner than the normative database due to segmentation artefact. [18] Not all cases of ERM were appreciated clinically on the time of recruitment as the vision was better than 6/18 in each eye. Myopia represents another challenge in glaucoma diagnosis, as both myopia and glaucoma independently cause thinning of the cRNFL and macular GCIPL.[19] Patients with myopia could also present with anomalous discs that affect the structural assessment of RNFL, such as tilting and peripapillary atrophy, or posterior myopic staphyloma. Myopic staphyloma is an outward fundus excavation around the disc or posterior pole, leading to distortion of the retinal structure and a concave appearance on the B-scan OCT [9].

In conclusion, almost 94% of the baseline GCA scans of the PROGRESSA cohort were without error; therefore, GCA could be considered a valid method of assessment. This agrees with recent studies reporting high reproducibility of GCIPL thickness measurements using the Cirrus HD-OCT, [20, 21] and high efficacy for detecting early glaucomatous structural change. [2, 22] Evidence supports the use of this imaging modality by appropriately trained professionals, as a diagnostic adjunct to clinical examination. However, failure to appreciate scan artefacts can lead to misinterpretation of the results. The rate of co-morbid macular pathology and scan artefact is likely to be higher in clinical practice than what we have reported, since scans were acquired by highly experienced operators and the PROGRESSA cohort excluded individuals with poor visual acuity and non-glaucomatous visual field defects.

## Supporting information

S1 Table Eye characteristics of the patients associated with ganglion cell layer and inner plexiform layer scan artefacts. (PDF)

## References

[1] Na JH, Sung KR, Baek S, Sun JH, Lee Y. Macular and retinal nerve fiber layer thickness: which is more helpful in the diagnosis of glaucoma? Invest Ophthalmol Vis Sci. 2011;52(11):8094–101. 10.1167/iovs.11-7833 21911590 10.1167/iovs.11-7833

[2] Mwanza JC, Durbin MK, Budenz DL, Sayyad FE, Chang RT, Neelakantan A, et al Glaucoma diagnostic accuracy of ganglion cell-inner plexiform layer thickness: comparison with nerve fiber layer and optic nerve head. Ophthalmology. 2012;119(6):1151–8. 10.1016/j.ophtha.2011.12.014 22365056 10.1016/j.ophtha.2011.12.014

[3] Kotowski J, Folio LS, Wollstein G, Ishikawa H, Ling Y, Bilonick RA, et al Glaucoma discrimination of segmented cirrus spectral domain optical coherence tomography (SD-OCT) macular scans. Br J Ophthalmol. 2012;96(11):1420–5. 10.1136/bjophthalmol-2011-301021 22914498 10.1136/bjophthalmol-2011-301021 PMC3721629

[4] Mitchell P, Smith W, Chey T, Wang JJ, Chang A. Prevalence and associations of epiretinal membranes. The Blue Mountains Eye Study, Australia. Ophthalmology. 1997;104(6):1033–40. 9186446 10.1016/s0161-6420(97)30190-0

[5] Leung CK, Ye C, Weinreb RN, Yu M, Lai G, Lam DS. Impact of age-related change of retinal nerve fiber layer and macular thicknesses on evaluation of glaucoma progression. Ophthalmology. 2013;120(12):2485–92. 10.1016/j.ophtha.2013.07.021 23993360 10.1016/j.ophtha.2013.07.021

[6] Mwanza JC, Oakley JD, Budenz DL, Chang RT, Knight OJ, Feuer WJ. Macular ganglion cell-inner plexiform layer: automated detection and thickness reproducibility with spectral domain-optical coherence tomography in glaucoma. Invest Ophthalmol Vis Sci. 2011;52(11):8323–9. 10.1167/iovs.11-7962 21917932 10.1167/iovs.11-7962 PMC3208140

[7] Hodapp E, Parrish RK, Anderson DR. Clinical Decisions in Glaucoma St Louis, Mo: Mosby; 1993 P52–61

[8] Spaeth GL, Henderer J, Liu C, Kesen M, Altangerel U, Bayer A, et al The disc damage likelihood scale: reproducibility of a new method of estimating the amount of optic nerve damage caused by glaucoma. Trans Am Ophthalmol Soc. 2002;100:181–5; discussion 5–6. 12545692 PMC1358961

[9] Faghihi H, Hajizadeh F, Riazi-Esfahani M. Optical coherence tomographic findings in highly myopic eyes. J Ophthalmic Vis Res. 2010;5(2):110–21. 22737340 PMC3380683

[10] Mwanza JC, Budenz DL, Godfrey DG, Neelakantan A, Sayyad FE, Chang RT, et al Diagnostic performance of optical coherence tomography ganglion cell—inner plexiform layer thickness measurements in early glaucoma. Ophthalmology. 2014;121(4):849–54. 10.1016/j.ophtha.2013.10.044 24393348 10.1016/j.ophtha.2013.10.044

[11] Nouri-Mahdavi K, Nowroozizadeh S, Nassiri N, Cirineo N, Knipping S, Giaconi J, et al Macular ganglion cell/inner plexiform layer measurements by spectral domain optical coherence tomography for detection of early glaucoma and comparison to retinal nerve fiber layer measurements. Am J Ophthalmol. 2013;156(6):1297–307 e2. 10.1016/j.ajo.2013.08.001 24075422 10.1016/j.ajo.2013.08.001 PMC3834195

[12] Han IC, Jaffe GJ. Evaluation of artifacts associated with macular spectral-domain optical coherence tomography. Ophthalmology. 2010;117(6):1177–89 e4. 10.1016/j.ophtha.2009.10.029 20171740 10.1016/j.ophtha.2009.10.029

[13] Asrani S, Essaid L, Alder BD, Santiago-Turla C. Artifacts in spectral-domain optical coherence tomography measurements in glaucoma. JAMA Ophthalmol. 2014;132(4):396–402. 10.1001/jamaophthalmol.2013.7974 24525613 10.1001/jamaophthalmol.2013.7974

[14] Liu Y, Simavli H, Que CJ, Rizzo JL, Tsikata E, Maurer R, et al Patient characteristics associated with artifacts in Spectralis optical coherence tomography imaging of the retinal nerve fiber layer in glaucoma. Am J Ophthalmol. 2015;159(3):565–76 e2. 10.1016/j.ajo.2014.12.006 25498118 10.1016/j.ajo.2014.12.006 PMC4423408

[15] Anwar Z, Wellik SR, Galor A. Glaucoma therapy and ocular surface disease: current literature and recommendations. Curr Opin Ophthalmol. 2013;24(2):136–43. 10.1097/ICU.0b013e32835c8aba 23542350 10.1097/ICU.0b013e32835c8aba

[16] Stein DM, Wollstein G, Ishikawa H, Hertzmark E, Noecker RJ, Schuman JS. Effect of corneal drying on optical coherence tomography. Ophthalmology. 2006;113(6):985–91. 10.1016/j.ophtha.2006.02.018 16751039 10.1016/j.ophtha.2006.02.018 PMC1933491

[17] Kim NR, Lee H, Lee ES, Kim JH, Hong S, Je Seong G, et al Influence of cataract on time domain and spectral domain optical coherence tomography retinal nerve fiber layer measurements. J Glaucoma. 2012;21(2):116–22. 10.1097/IJG.0b013e31820277da 21173702 10.1097/IJG.0b013e31820277da

[18] Lee HJ, Kim MS, Jo YJ, Kim JY. Thickness of the Macula, Retinal Nerve Fiber Layer, and Ganglion Cell Layer in the Epiretinal Membrane: The Repeatability Study of Optical Coherence Tomography. Invest Ophthalmol Vis Sci. 2015;56(8):4554–9. 10.1167/iovs.15-16949 26200495 10.1167/iovs.15-16949

[19] Rauscher FM, Sekhon N, Feuer WJ, Budenz DL. Myopia affects retinal nerve fiber layer measurements as determined by optical coherence tomography. J Glaucoma. 2009;18(7):501–5. 10.1097/IJG.0b013e318193c2be 19745664 10.1097/IJG.0b013e318193c2be PMC2742764

[20] Francoz M, Fenolland JR, Giraud JM, El Chehab H, Sendon D, May F, et al Reproducibility of macular ganglion cell-inner plexiform layer thickness measurement with cirrus HD-OCT in normal, hypertensive and glaucomatous eyes. Br J Ophthalmol. 2014;98(3):322–8. 10.1136/bjophthalmol-2012-302242 24307717 10.1136/bjophthalmol-2012-302242

[21] Ng DS, Gupta P, Tham YC, Peck CF, Wong TY, Ikram MK, et al Repeatability of Perimacular Ganglion Cell Complex Analysis with Spectral-Domain Optical Coherence Tomography. J Ophthalmol. 2015;2015:605940 10.1155/2015/605940 26229687 10.1155/2015/605940 PMC4502302

[22] Choi YJ, Jeoung JW, Park KH, Kim DM. Glaucoma detection ability of ganglion cell-inner plexiform layer thickness by spectral-domain optical coherence tomography in high myopia. Invest Ophthalmol Vis Sci. 2013;54(3):2296–304. 10.1167/iovs.12-10530 23462754 10.1167/iovs.12-10530

