# Supplementary material for: Prevalence and type of artefact with spectral domain optical coherence tomography macular ganglion cell imaging in glaucoma surveillance
Source: PLoS One. 2018 Dec 5;13(12):e0206684. doi: 10.1371/journal.pone.0206684 (PMC6281246; doi:10.1371/journal.pone.0206684)
Supplement: S1 Table — (PDF) [file pone.0206684.s001.pdf]

Supplementary table 1: Eye characteristics of the patients associated with ganglion cell layer and inner plexiform layer scan artefacts.

EMG: early manifest glaucoma

Eye ID	Age of the patient in years	Glaucoma status Condition	Detected GCIPL arefacts
P01	66	Glaucoma Suspect	Acquisition error
P02	70	EMG	Myopic error
P03	80	EMG	Vitreo-Macular interface abnormality
P04	61	Glaucoma Suspect	Machine segmentation error
P05	69	EMG	Myopic error
P06	86	Glaucoma Suspect	Vitreo-Macular interface abnormality
P07	80	Glaucoma Suspect	Myopic error
P08	80	Glaucoma Suspect	Floaters
P09	81	Glaucoma Suspect	Floaters
P10	71	Glaucoma Suspect	Vitreo-Macular interface abnormality
P11	71	Glaucoma Suspect	Myopic error
P12	72	Glaucoma Suspect	Myopic error
P13	81	Glaucoma Suspect	Macular schesis
P14	78	Glaucoma Suspect	Signal strength <6/10
P15	83	Glaucoma Suspect	Signal strength <6/10
P16	70	EMG	Myopic error
P17	72	EMG	Machine segmentation error
P18	77	EMG	Machine segmentation error
P19	82	EMG	Machine segmentation error
P20	76	EMG	ERM
P21	62	Glaucoma Suspect	Floaters
P22	87	Glaucoma Suspect	Vitreo-Macular interface abnormality
P23	63	Glaucoma Suspect	Machine segmentation error
P24	88	EMG	Floaters
P25	72	Glaucoma Suspect	Floaters
P26	67	EMG	ERM
P27	79	EMG	Floaters
P28	77	EMG	Myopic error
P29	67	Glaucoma Suspect	ERM
P30	72	EMG	ERM/Myopic error
P31	80	Glaucoma Suspect	ERM
P32	84	Glaucoma Suspect	ERM
P33	82	Glaucoma Suspect	Machine segmentation error
P34	58	Glaucoma Suspect	Myopic eror
P35	74	EMG	Machine segmentation error
P36	63	EMG	Machine segmentation error
P37	54	Glaucoma Suspect	ERM
P38	82	EMG	ERM
P39	73	EMG	Signal strength <6/10

P40	61	EMG	Macular schesis
P41	72	EMG	ERM/Myopic error
P42	67	Glaucoma Suspect	ERM/Myopic error
P43	57	Glaucoma Suspect	ERM
P44	81	Glaucoma Suspect	Floaters
P45	78	Glaucoma Suspect	ERM
P46	77	Glaucoma Suspect	ERM
P47	82	EMG	Signal strength <6/10
P48	68	EMG	ERM
P49	80	EMG	ERM
P50	71	Glaucoma Suspect	ERM
P51	86	Glaucoma Suspect	Signal strength <6/10
P52	73	EMG	Floaters
P53	74	EMG	ERM
P54	65	Glaucoma Suspect	Machine segmentation error
P55	68	Glaucoma Suspect	ERM
P56	66	Glaucoma Suspect	ERM
P57	68	EMG	ERM
P58	80	Glaucoma Suspect	ERM
P59	75	EMG	Floaters
P60	72	EMG	Macular schesis
P61	79	Glaucoma Suspect	Signal strength <6/10
P62	70	EMG	Signal strength <6/10
P63	73	Glaucoma Suspect	Signal strength <6/10
P64	69	Glaucoma Suspect	Floaters
P65	61	Glaucoma Suspect	ERM/Myopic error
P66	71	EMG	ERM
P67	69	EMG	ERM
P68	66	Glaucoma Suspect	ERM
P69	70	Glaucoma Suspect	Acquisition error
P70	70	EMG	Signal strength <6/10
P71	70	Glaucoma Suspect	Vitreo-Macular interface abnormality
P72	81	Glaucoma Suspect	ERM
P73	67	Glaucoma Suspect	Machine segmentation error
P74	55	Glaucoma Suspect	Signal strength <6/10
P75	45	EMG	ERM
P76	75	EMG	AMD
P77	79	Glaucoma Suspect	ERM
P78	79	Glaucoma Suspect	Macular schesis
P79	59	Glaucoma Suspect	ERM
P80	54	EMG	Machine segmentation error
P81	74	EMG	ERM
P82	75	EMG	Floaters
P83	87	EMG	Machine segmentation error
P84	81	EMG	AMD

P85	74	Glaucoma Suspect	Signal strength <6/10
P86	90	EMG	ERM
P87	59	Glaucoma Suspect	ERM
